# Supplementary material for: Long Chain N3-PUFA Decreases ACE2 Protein Levels and Prevents SARS-CoV-2 Cell Entry
Source: Int J Mol Sci. 2022 Nov 10;23(22):13825. doi: 10.3390/ijms232213825 (PMC9695276; doi:10.3390/ijms232213825)
Supplement: Supplementary file 1 [file ijms-23-13825-s001.zip › ijms-1868182-supplementary.pdf]

**SUPPLEMENTARY FIGURES:**

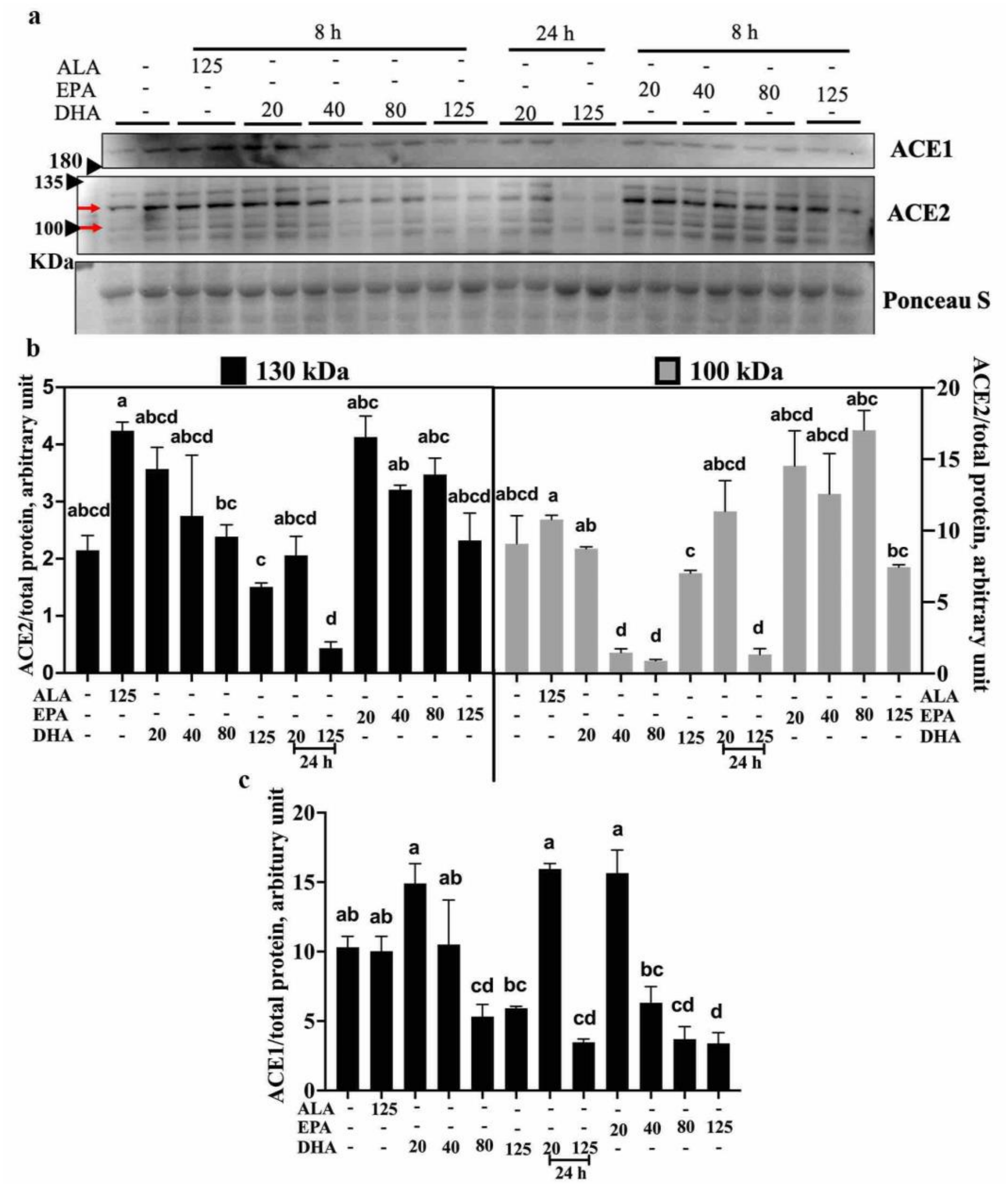


**Figure S1. DHA reduced both ACE2 and ACE1 levels in HEK 293 cells**

(a) Representative Western blots showing ACE1 and ACE2 levels relative to total protein as measured by Ponceau S in HEK 293 cells treated with n3-PUFA as indicated. The band intensities of ACE2 and ACE1 were quantified and are graphically presented in panels (b) and (c), respectively, normalized to the Ponceau staining. The 100 kDa band (grey bars) is native ACE2, while the 130 kDa band (black bars) is N-glycosylated ACE2. Unit of concentration for n3-PUFA treatments: μM. Data are presented as mean ± SEM, n = 4; in the graphs, bars not sharing a common letter are significantly different ($p < 0.05$) based on Duncan's Multiple range or LSD post-hoc tests.
